# Vegetation dynamics in Alpine glacier forelands tackled from space

**DOI:** 10.1038/s41598-019-50273-2

**Published:** 2019-09-26

**Authors:** Andrea Fischer, Thomas Fickert, Gabriele Schwaizer, Gernot Patzelt, Günther Groß

**Affiliations:** 1 Institute for Interdisciplinary Mountain Research, Austrian Academy of Sciences, Technikerstr. 21a, 6020 Innsbruck, Austria; 2 Faculty of Arts and Humanities, University of Passau, Innstraße 40, 94032 Passau, Germany; 3 ENVEO IT GmbH, Fürstenweg 176, 6020 Innsbruck, Austria; 4 Patscher Strasse 20, 6080 Igls, Austria; 5 Oberrain 205, 6721 Thüringerberg, Austria

**Keywords:** Climate-change ecology, Environmental sciences, Cryospheric science

## Abstract

Monitoring of plant succession in glacier forelands has so far been restricted to field sampling. In this study, *in situ* vegetation sampling along a chronosequence between Little Ice Age (LIA) maximum extent and the recent glacier terminus at Jamtalferner in the Austrian Alps is compared to time series of the Normalized Difference Vegetation Index (NDVI) calculated from 13 Landsat scenes (1985–2016). The glacier terminus positions at 16 dates between the LIA maximum and 2015 were analysed from historical maps, orthophotos and LiDAR images. We sampled plots of different ages since deglaciation, from very recent to approx. 150 years: after 100 years, roughly 80% of the ground is covered by plants and ground cover does not increase significantly thereafter. The number of species increases from 10–20 species on young sites to 40–50 species after 100 years. The NDVI increases with the time of exposure from a mean of 0.11 for 1985–1991 to 0.20 in 2009 and 0.27 in 2016. As the increase in ground cover is clearly reproduced by the NDVI (R² ground cover/NDVI 0.84) – even for sparsely vegetated areas –, we see a great potential of satellite-borne NDVI to perform regional characterizations of glacier forelands for hydrological, ecological and hazard management-related applications.

## Introduction

Geochronological records enhance our understanding of changes in both anthropogenic and environmental systems^1^. They comprise data on past vegetation and land use, which contribute to refining future adaptation processes. Dendrochronological evidence from glacier tongues is among the most prominent records, documenting the growth of trees, the controlling environmental conditions and the date of their death by overrunning ice. This paper aims to improve our understanding of the environmental conditions and the pace and delays in biotic response to changes in climate and glaciation by introducing and validating a new method to map biotic succession on a regional scale. This will improve the study of glaciological dendrochronological records by estimating the pace of past plant growth. So far, field data on plant succession, i.e. ground cover, species composition, vegetation structure, and its development over time are rather localized. Time series of glacier inventories from LIA onwards are rare. Thus, it would be a tremendous improvement in our knowledge of glacier changes if the LIA maximum, and maybe even retreat rates, could be compiled from biotic indicators, in particular in regions where maps are few or other information is sparse.

The current rapid glacier retreat^1^ opens up a perfect setting for studying succession under rapidly warming conditions. Austria’s glaciers have retreated since their LIA maximum extent in the middle of the 19^th^ century, losing more than half their former area^2^. Retreat rates varied during this period, including two short periods of glacier advances in the 1920s and 1980s. During the last decade, Alpine glaciers have retreated at an historically unprecedented rate^3^. The glacier retreat modifies the hydrological response and the sediment transport within glacier-covered basins and subsequently destabilizes the paraglacial area^4^. Forecast and mitigation of the downstream effects are considered essential^5,6^. Glacier retreat also opens up new ground for the succession of biota, stabilizing the paraglacial terrain and allowing storage and interception of precipitation. Whatever the future glacier scenarios, which range from total loss of glaciers within the next decades^7^ to more moderate scenarios^8^, the pace and amount of biotic succession will be key for developing climate change adaptation measures in high Alpine settlements in glacier-covered basins. For the interpretation of dendrochronological and other biotic paleodata in glacier forelands, knowledge of the time required for different species and/or life forms to develop is an important constraint.

Motivated by these two facts, the study at hand investigates a chronosequence in the paraglacial glacier foreland of Jamtalferner in the Austrian part of the Silvretta to compile a data base for further analysis of the glacier-climate-plant succession interactions at the site and use this data base to verify the results of a remote-sensing approach and to pave the ground for adding further sites to a regional data base.

**Figure 1:**
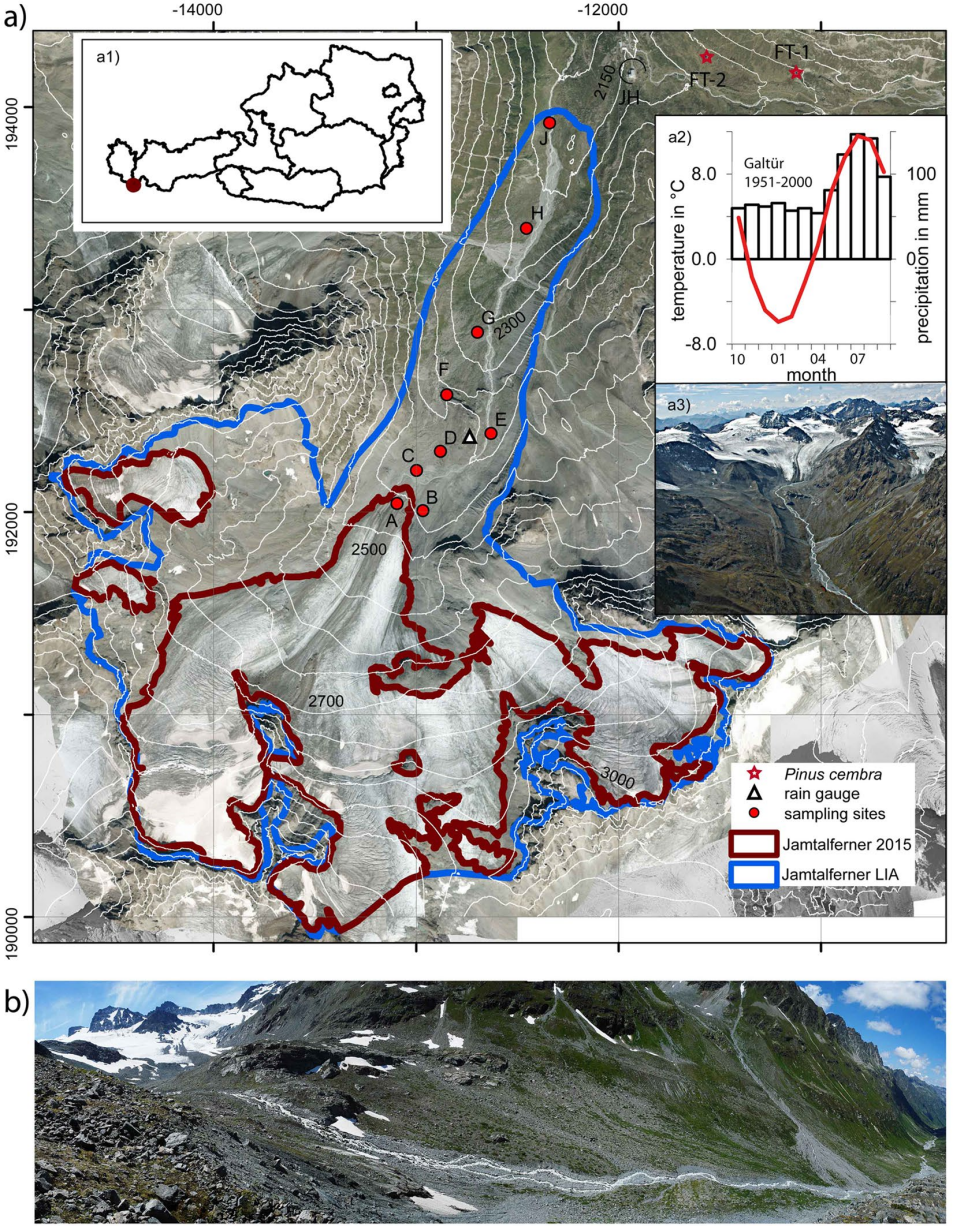
(**a**) Extent of Jamtalferner at LIA maximum (blue line) and in 2015 (red line) as well as contour lines (DEM of 2006; source: Land Tirol - data.tirol.gv.at CC BY 4.0) superimposed on an orthophoto from August 2015. Red dots indicate vegetation sampling sites A to J in 2016. The three subsites (I, II and III) for each sample location are close to each other, so that only one symbol is displayed at the averaged positions of the subsites. JH indicates the location of Jamtal hut, FT1 and FT2 indicate the discoveries of fossil *Pinus cembra* trees. Inserts: (a1) location of Jamtalferner within Austria, (a2) mean monthly temperatures and precipitation at the station of Galtür, and a3) photo of the upper part of Jamtalferner (2006, A. Fischer). (**b**) Panoramic view of the glacier foreland of Jamtalferner between the current and the LIA terminus (2018, A. Fischer).

To tackle the influence of biota on the consolidation of paraglacial areas and to gain information on past retreat rates, remote sensing would be the perfect tool to derive information on a regional scale, for areas difficult to access, and at high repeat rates. The high spatial resolution of optical satellite-borne imagery available today, with repeat rates from just a few days to weeks, might prepare the ground for new applications in the current phase of glacier decay, with rapidly growing paraglacial areas. This multidisciplinary study contributes to these aims by providing ground truth of glacier area changes, together with the evolving biotic succession, in a well-studied area, the Jamtalferner glacier basin. The Jamtal is located in the Eastern Alps in the Austrian part of the Silvretta mountain range (Fig. 1). The highest peak at the watershed surrounding the Jamtalferner is the Dreiländerspitze (3197 m a.s.l.). Jamtalferner is one of Austria’s larger glaciers, terminating in a flat tongue flowing towards NNE and fed by three firn tributaries (Fig. 1). The Jamtal ends at the village of Galtür (1587 m a.s.l., 10.1858°E, 46.9728°N, about 11 km from the glacier tongue in 2015), where a climate station of the national weather service ZAMG is located. Monthly and seasonal means of air temperature and sums of precipitation are shown in Table S1 for the period from 1951 to 2000. In addition, a homogenized time series of monthly mean air temperatures at the station at Galtür is available from the HISTALP data set^9^ up to 2015 and monthly means measured at Galtür provided by the Hydrographical Service of the Federal Government of Tyrol^10^ thereafter. The general climate of the Silvretta is (sub)continental due to rain shadow effects caused by the Northern Limestone Alps. Summit areas are humid year-round, receiving a mean annual precipitation between 1200 and 1600 mm^11^. At the glacier tongue, a mean of 1507 mm of precipitation was recorded for the period 1989–2017. Most of the high elevation precipitation falls as snow, resulting in a long-lasting snow cover, roughly from December to May. Assuming a vertical temperature lapse rate of 0.6 °C/100 m, a mean annual temperature of −2.5 °C can be assumed at the recent glacier terminus. Geologically, the Jamtal is located within the Silvretta nappe, belonging to the crystalline Eastern Alpine tectonic unit. Metamorphic rocks prevail, mostly different types of gneiss or amphibolite. Substrate in the glacier foreland of Jamtalferner is coarse-grained and blocky siliceous glacial debris. Within the recently deglaciated areas there are no visible signs of soil development, while in areas deglaciated during the decades following LIA maximum extent, syrosems and rankers are present. Current land use is by cattle husbandry during the summer months, but the area has been subject to anthropogenic use for several millennia^12^. Analysis of pollen^13^ and radiocarbon-dated fossil *Pinus cembra* trees^14^ found close to the Jamtal hut (Fig. 1, Table S2) document the alteration of vegetation, including the tree line, as a result of both land use and climatic changes in the past.

For the Jamtal glacier basin, maps of the size and position of glaciers are available from 1774 onwards. From earlier maps we can learn of hydrological features besides the glaciation. The glacial river today flows in the central part of the former glacier bed, with a sander close to the current glacier terminus. Between the LIA terminus and the sander (about 1.5 km from the LIA terminus) the glacier bed significantly changes its slope because of a steep rocky outcrop. The glacier retreated from this rocky outcrop between approximately 1949 and 1954. Behind the rocky outcrop, the slope inclines only very gently, as far as visible until 2018. The first installation of a length change monitoring network took place in 1892, and glacier mass balance monitoring started in 1988/89^10,15^. Paleo-glaciological^16,17^ and archaeological studies^12^ provide evidence of paleo treelines. In 2016 a chronosequence on vegetation development was carried out at nine locations of different site ages between the LIA moraines and the current (2016) glacier terminus, a horizontal distance of about 2 km. Optical remote-sensing data (Landsat) are available from 1972 onwards. As the spectral and spatial resolution of Landsat 1 images is limited, compared to later missions, our time series of Landsat data start in 1985. By means of maps and orthophotos, the position of the glacier terminus was compiled for 16 points in time between LIA maximum and 2015, allowing for a quite precise estimate of the date of deglaciation for particular areas as well as for the site age of vegetation samples of the chronosequence. Historical photographs support the analysis and provide additional information on changes in the fluvial system in the paraglacial area. The Normalized Difference Vegetation Index (NDVI) is calculated from a time series of remote-sensing data and linked to ground cover values and vegetation structure at the sample sites to test whether the recorded increase in vegetation over time could be reproduced by remote-sensing data. As high variability in both glacier retreat and succession rates, as well as erratic disturbances that push succession back to earlier stages, can be expected between different glacier forelands, the proposed method needs further validation.

## Results

### Historical area and length changes of Jamtalferner

**Figure 2:**
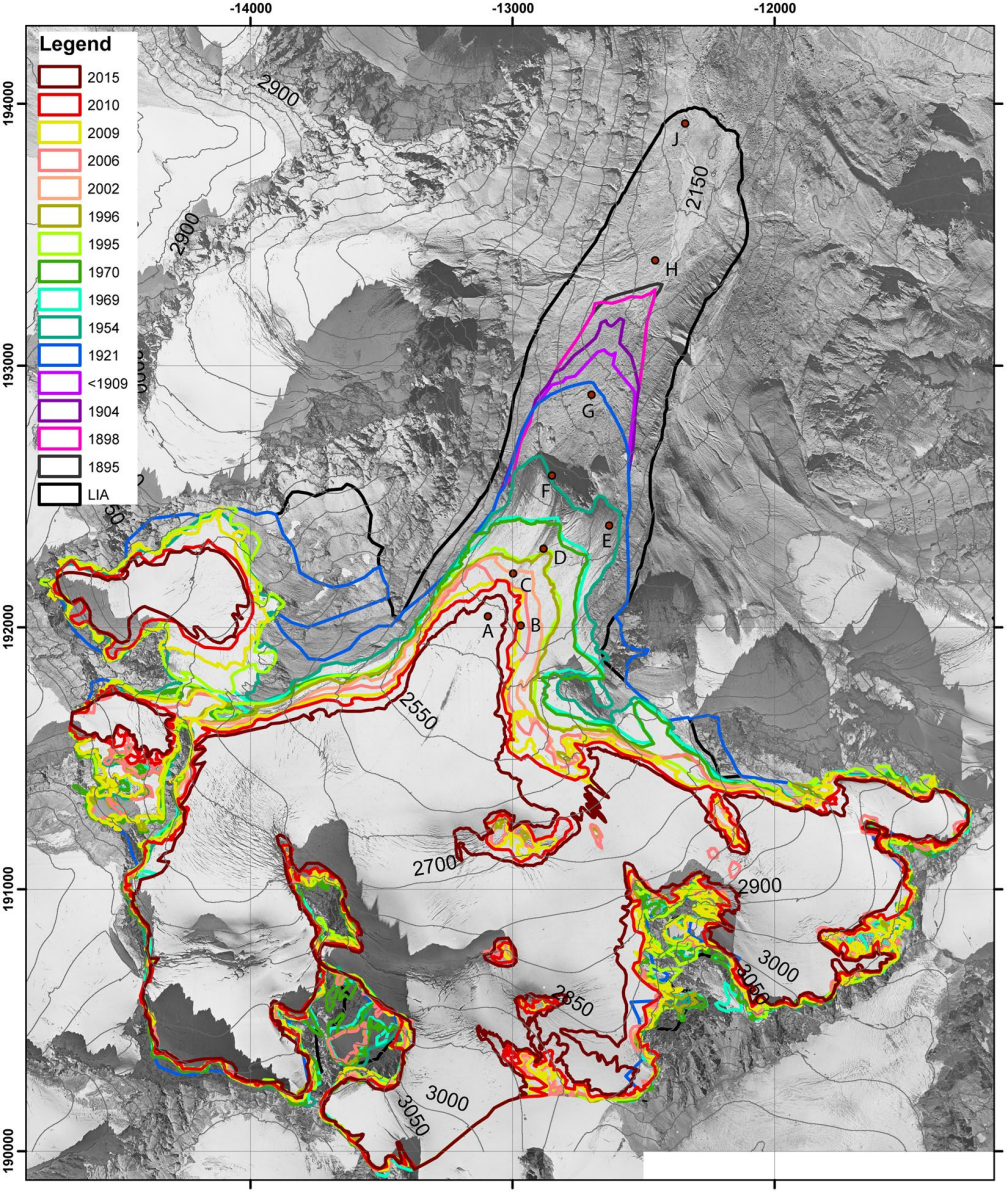
Glacier outlines mapped from the sources listed in Table S6 with vegetation sampling locations A-J. The three subsites (I, II and III) for each sample location are close to each other, so that only one symbol is displayed at the averaged positions of the subsites. Background: orthophoto 1954 (source: Land Tirol - data.tirol.gv.at CC BY 4.0).

The area covered by Jamtalferner shrank by 53.4% between the LIA maximum in 1864 and the year 2015 (Table 1, Figs 1 and 2). The mean annual rates of area loss were below 1% for all periods prior to 2006, except for the year 1996, and exceeded 1% thereafter. The elevation of the lowest point of Jamtalferner has risen from 2120 m a.s.l. (in the 1860s) to 2406 m a.s.l. in 2006 (for later periods, no DEM is available). Until about the year 2000, a major part of the area changes took place close to the glacier terminus. In the 21^st^ century, however, area loss has also affected higher parts of the glacier, with the disintegration of the three major tributaries and nunataks standing proud in the former main areas of the glacier. Both area and length changes are results of mass balance, glacier dynamics, ice thickness and bedrock geometry.

**Table 1:** Area changes at Jamtalferner for the period 1864–2018, with annual area losses and lowest glacier altitudes in the periods from direct measurements and calculated from the shapes (GIS). *… no DEM of the glacier bed is available.

Year	Interval (in years) to previous measurement	Area		Annual area loss		Lowest glacier altitude
	years	km²	% of LIA maximum	km²/y	%/y	m a.s.l.
1864		6.041	###			2120
1895	31	5.252	86.9	−0.025	−0.4	2175
1898	3	5.247	86.9	−0.002	0.0	2178
1904	6	5.208	86.2	−0.006	−0.1	2202
1909	5	5.174	85.6	−0.007	−0.1	2236
1921	12	5.136	85.0	−0.003	−0.1	2236
1954	33	4.531	75.0	−0.018	−0.3	2262
1969	15	4.141	68.6	−0.026	−0.4	2397
1970	1	4.115	68.1	−0.026	−0.4	2398
1995	25	3.896	64.5	−0.009	−0.1	2403
1996	1	3.808	63.0	−0.088	−1.5	2403
2002	6	3.682	61.0	−0.021	−0.3	2404
2006	4	3.571	59.1	−0.028	−0.5	2406
2009	3	3.375	55.9	−0.065	−1.1	*
2010	1	3.207	53.1	−0.168	−2.8	*
2015	5	2.818	46.6	−0.078	−1.3	*
Total	151

**Figure 3:**
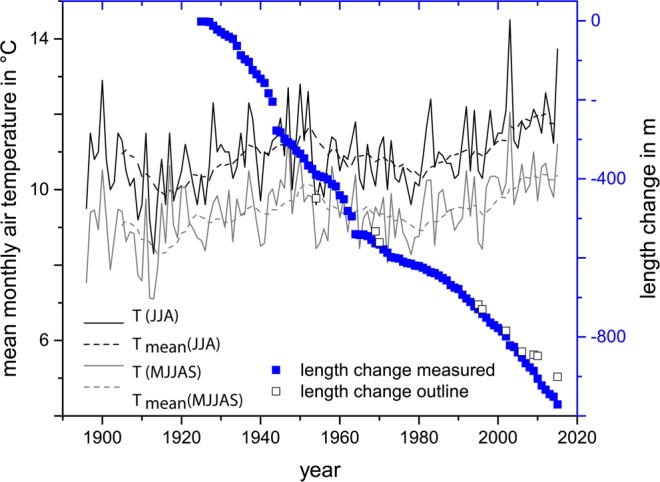
Length change records of Jamtalferner provided by the Austrian Alpine Club (full blue squares), and length changes along the central flowline from the glacier outlines (unfilled squares) plotted against the air temperatures (T) of the weather station in Galtür: T(JJA) mean of the summer months June, July and August, T(MJJAS) mean of the ablation period May to September). The running decadal means are denoted T_mean_.

The glacier length changes have been measured by observers of the Austrian Alpine Club^18^. The first observer, Ignaz Lorenz, installed reference marks in 1892. Current data compilations include annual values from 1925 onwards. In the few cases when measurements were only taken once every few years (e.g. between 1913 and 1924 length change was not measured by the observers of the Alpine Club), the measured change was attributed evenly across the missing years as steady retreat rates. The length change records present a continuous retreat of the glacier tongue. In 1924, H. Kinzl, in an unpublished report, described the existence of a small discontinuous moraine from a glacier advance, which was also mapped by Vorndran^19^. Greim^20^ reported the stagnation of the retreat for 1915 and an elevation increase of the profiles at the surface of the glacier tongue, compared to measurements in 1895/97, which would indicate increasing volume/flow velocities, which, however, were not large or long enough to trigger a glacier advance. High annual retreat rates were recorded between the mid-1930s and the mid-1960s, slowing down until the early 1990s, and reaching higher rates again afterwards. The reason for that is a cooler period from about 1950 to 1990 (Fig. 3).

The cumulative length change calculated from the records of the Austrian Alpine Club fits remarkably well with the total length change along the flow line as evident from the glacier outlines with less than 10% deviation (Table 2). Results from GIS analysis and direct measurements deviate less than 1% per annum in most periods. Exceptions are the very short periods 1969/1970, 2006–2009, 2009/2010, when the absolute differences of 24.8 m, 8.7 m and 16.6 m, respectively, may still be considered to be within the uncertainties of the measurements. The period 1954–1969 presents an annual relative deviation of 7.1%, resulting from an absolute difference of 106 m over 15 years. Taking into account all the uncertainties in measuring length changes and presuming a central flow line, the pace of glacier length change and thus the deglaciation of new ground available for plant succession is in high agreement for both methods.

**Table 2:** Length changes at Jamtalferner for the period 1864–2018, with annual area losses and lowest glacier altitudes in the periods from direct measurements and calculated from the shapes (GIS). *… no DEM of the glacier bed is available, #… direct length change measurements from 1925 onwards, GIS data refers to 1921.

Year	Interval (in years) to previous measurement	Length change	GIS		Total period	difference	Per year
		measured			difference		difference
	years	m	m		m	%	m
1864							
1895	31	784					
1898	3	30					
1904	6	95					
1909	5	137					
1921	12	135					
1954	33	449	389.5	#	59.5	15.3	1.8
1969	15	84	173.4		−89.4	−51.6	−6.0
1970	1	28	3.2		24.8	775.0	24.8
1995	25	157	163.7		−6.7	−4.1	−0.3
1996	1	12	12.1		−0.1	−0.8	−0.1
2002	6	54.8	57		−2.2	−3.9	−0.4
2006	4	52.6	53.6		−1	−1.9	−0.3
2009	3	7.6	33.8		−26.2	−77.5	−8.7
2010	1	2.8	19.4		−16.6	−85.6	−16.6
2015	5	53.2	65.4		−12.2	−18.7	−2.4
Total	151	901	971.1		−70.1	−7.2	−0.5

### Chronosequence

**Figure 4:**
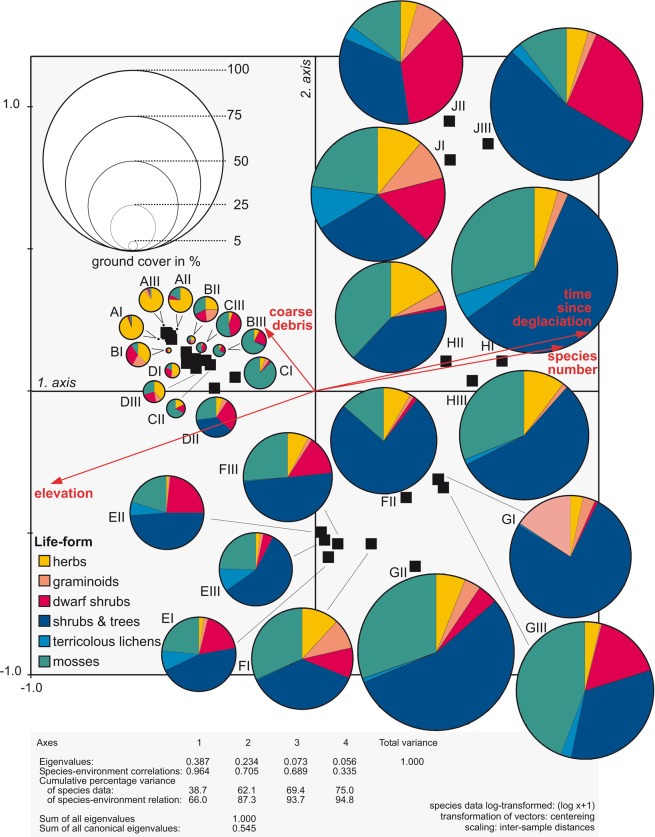
PCA scatter plot of the chronosequence samples in the glacier foreland of Jamtalferner, based on species composition to depict the floristic similarity between samples (the closer located the black sample-symbols within the ordination space, the higher the similarity). The superimposed pies indicate life form composition and total ground cover of the samples. Where necessary (A, B, and CIII), pies are zoomed for better reading.

**Figure 5:**
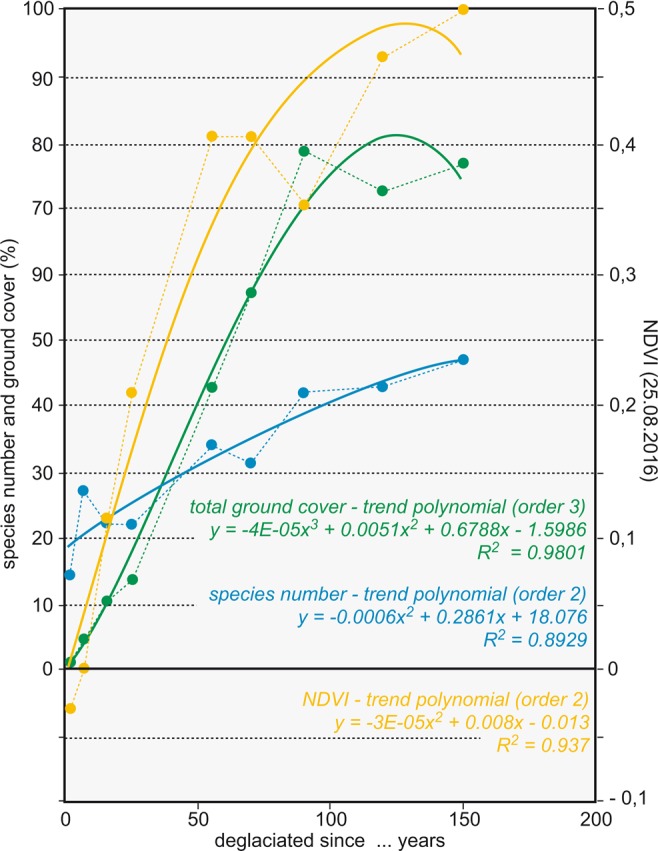
Development (mean out of three samples) of species numbers (blue), individual numbers (green), as well as NDVI values (yellow) along the chronosequence in the glacier foreland of Jamtalferner.

Along the studied chronosequence, a total of 82 vascular plant species, four terricolous lichens and mosses (as species group) were recorded (Table S3). All sample sites are variably blocky, with rocks of >6 cm edge length making up between less than 10% and 80%. The Principal Component Analysis (PCA) scatter plot in Fig. 4 indicates the floristic similarity between samples as well as changes in ground cover and life form composition during succession. As reported from other glacier forelands of the Alps, colonization of bare ground starts immediately after glacier retreat (e.g.^21–23^). Just one or two years after deglaciation (A-sites), a total of 13 different vascular plant species, predominately herbaceous taxa, as well as mosses were recorded. Most common early colonizers are *Epilobium anagallidifolium*, *Cerastium uniflorum* and *Gnaphalium supinum*. Ground cover, however, is very low, with a mean (out of three samples) of only 0.35% (Fig. 4, Table S3). On sites deglaciated for seven years (B-sites), the number of vascular plant species is doubled and also mean ground cover is considerably higher (4.24%), controlled, however, primarily by a high moss cover of almost 2% (Table S3). Life-form composition is more varied, with many different life forms co-occurring (Fig. 4). Despite lower total species numbers (20) on the C- and D-sites (deglaciated for 15 and 25 years, respectively) compared to the B-sites, ground cover continues to increase with site age (Figs 4 and 5), on the C-sites again mainly by mosses, on the D-sites primarily by vascular plants. Here the shrub *Salix hegetschweileri* and the dwarf-shrub *Saxifraga bryoides* achieve mean ground cover values of more than 2% (Table S3).

**Figure 6:**
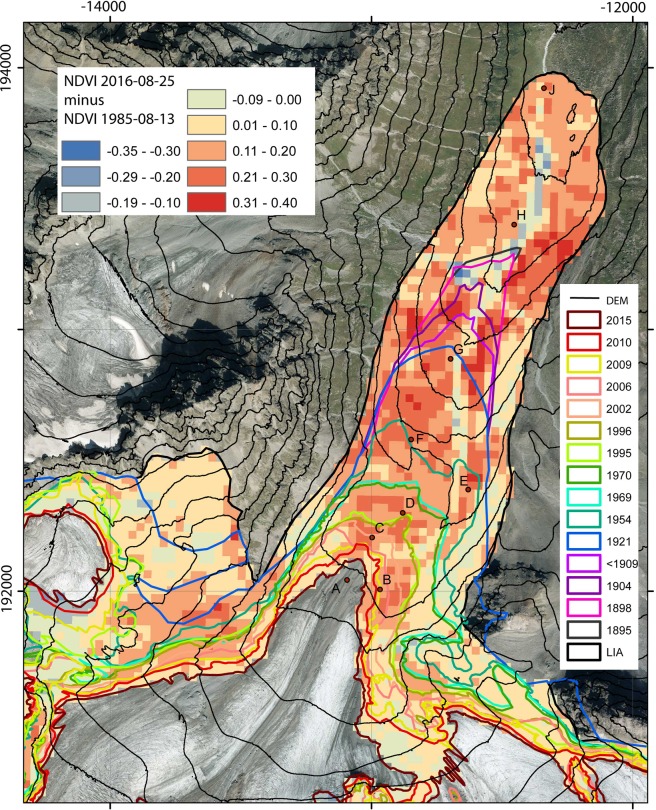
Map of NDVI difference between August 1985 and August 2016. Lowest values indicate regions with unstable ground (alluvial fans, river bed, unstable moraines), highest values indicate stable conditions with increasing vegetation cover. Orthophotos (source: Land Tirol - data.tirol.gv.at CC BY 4.0) of the same subset are found in the supplementary material (Figs S1 and S2).

Much higher species numbers and ground cover values are recorded on the E- and F-sites, deglaciated for 55 and 70 years, respectively (E: 32 vascular plant species, 42.8% mean ground cover; F: 29 vascular plant species, 57,3% mean ground cover). The shrub *Salix helvetica* is the most dominant species and provides half of the total ground cover. The trend of increasing ground cover and species numbers continues on sample sites G, H and J of at least 90 years after deglaciation (Figs 5 and 6, Table S3). Arranged rather discretely within the ordination space (Fig. 4), these stages are dominated by small trees, shrubs and mosses, with several woody taxa missing from earlier stages (e.g. *Rhododendron ferrugineum*, *Salix glaucosericea*, *Larix decidua*). On the oldest sites (J), ericaceous dwarf shrubs (*Vaccinium gaultherioides*, *V. myrtillus*, *Empetrum hermaphroditum*, *Calluna vulgaris*, *Loiseleuria procumbens*) become more prominent (Figs 4 and 5, Table S3).

### NDVI

**Figure 7:**
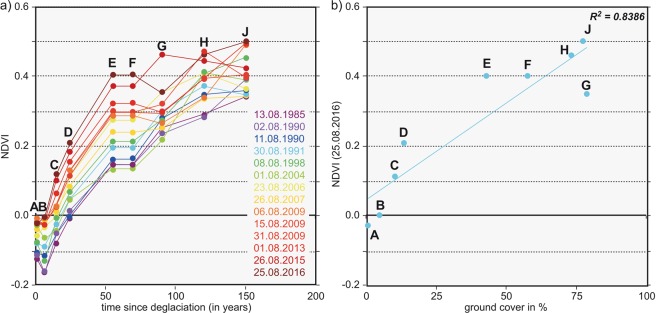
(**a**) NDVI in August of the years 1985–2016, calculated from snow-free clear-sky Landsat pixels at the location of the vegetation sampling sites (displayed value is a mean of the three sub-samples I, II and III). (**b**) Correlation of NDVI values with mean ground cover.

The mean of the three subsamples basically reveals an increase in plant cover with time since deglaciation for all remote-sensing images (Fig. 6, Table S4). The increase in NDVI is highest at sites E and F (ice-free for 55 and 70 years). The increase, however, is not constant, for example, the NDVI at sample site G is in some cases lower than that for sample sites E and F, which became deglaciated later than G. At most sample sites, NDVI increases with time since deglaciation and is highly correlated to ground cover (Fig. 7). The interannual variability varies from site to site, and in some remote-sensing images locations deviate from the general increase in NDVI, for instance, in the images of 26 August 2015 for sample site G. Nevertheless, between 1985 and 2016, the mean NDVI of all plots more than doubled. Changes in NDVI clearly indicate regions with stable and unstable ground (alluvial fans, river bed, unstable moraines, see Fig. 6), as the comparison with orthophotos of 1970 and 2015 (supplementary material, Figs S1 and S2) confirms. However, the patterns of NDVI changes over time (Fig. 6) still do not allow for any straightforward interpretation.

## Discussion and Conclusion

Early accounts of plant development in the glacier foreland of Jamtalferner date back to the late 19^th^ century^24,25^. Species reported for the area between the LIA terminal moraine and the ice margin at that time, i.e. for the first decades after deglaciation, include *Ranunculus glacialis*, *Linaria alpina*, *Arabis alpina*, *Leucanthemopsis* (=*Chrysanthemum) alpinum*, *Achillea atrata*, *Hieracium alpinum*, *Salix herbacea*, *Saxifraga biflora*, *S. aspera*, *Saussurea alpina*, *Silene exscapa* (=*S. acauli*s), as well as several grasses, primarily of the genera *Poa* and *Festuca*. Those species were common early colonizers elsewhere in the eastern Alps after LIA maximum^26^ and they still are today: several of the species mentioned by^24^ for the post-LIA glacier foreland are also encountered today in the early stages of plant succession in the recently deglaciated glacier foreland of the Jamtalferner (see Table S3). However, because of the small stature of the plants and the low ground cover, neither maps nor photographs are able to depict vegetation development at these early stages of primary succession, thus failing as a source of information.

As recently shown for the glacier foreland of Lenksteinferner (South Tyrol), primary succession on deglaciated ground after LIA does not differ much from that occurring today, as thermal conditions within the glacier forelands after the LIA (at lower elevations but under colder climate) and the current ones (at higher elevation but affected by climate warming) are virtually identical^22^. Interestingly, some of the taxa reported from the glacier foreland of Jamtalferner for the late 19^th^ century^24,25^ are still present today within the same area, illustrating a high persistence of particular taxa once established (e.g. *Leucanthemopsis alpina*, *Salix herbacea*). This supports the assumption that not only pioneer species sensu stricto (i.e., early colonizers not able to persist over time during succession) are able to colonize the bare ground after ice retreat, but so are early- to late-successional taxa (i.e., those that persist over time during succession but are also early colonizers), if available in the surroundings^22^. Anyway, most species exhibit particular temporal abundancies within the successional development, allowing for the designation of successional stages: a pioneer stage (approximately one to two decades after deglaciation; sites A and B) with low ground cover despite a rather high number of different plant species (Fig. 5), which, however, occur with few individuals only. Substrate is coarse-grained and soil development totally absent. Which species colonize is highly random and depends on site conditions and seed sources in the surroundings. During an early successional stage (represented by sites C and D deglaciated for 15 and 25 years, respectively), ground cover rises to mean values roughly between ten and 15%, Figs 4 and 5). In particular on the C- sites mosses contribute much to total ground cover. Sites E to J are late successional with much higher ground cover and species numbers compared to the pioneer and early successional stage. Some of the early colonizing species disappear (e.g. *Epilobium anagallidifolium*, *Saxifraga exarata* ssp. *exarata*, *Silene exscapa*, *Arabis alpina*), while others are still present and joined by several additional taxa. Species numbers total between 30 and close to 50, and in particular shrubs contribute much to ground cover in the late successional stage (Fig. 4). Ground cover rises from 40–60% on sites E and F to around 80% on older sites (G-J). Sites E and F, deglaciated for 55 and 70 years, respectively, are somewhat transitional between the early successional stage and sites deglaciated for roughly a century or more concerning ground cover values and species composition (Table S3, Fig. 4). This is also expressed by the location of site FII within the ordination space (Fig. 4), indicating a floristic similarity to GI and GII, with many common species and esp. a high cover values of *Salix glaucosericea* (26.3%) compared to the other F- and the E-sites. Shrubs and trees also shape the older sites of the late successional stage (sites G-J, deglaciated for 90 years and more). There are several additional shrub species present that are missing on younger sites, and ericaceous shrubs and dwarf-shrubs (*Rhododendron ferrugineum*, *Vaccinium gaultherioides*, *V. myrtillus*, *Empetrum hermaphroditum, Loiseleuria procumbens*) are particularly important components on the oldest sites. In addition, coniferous tree species (*Larix decidua*, *Picea abies*) are sporadically present on sites deglaciated for more than one century. Although the altitude of the fossil remnants of *Pinus cembra* found close to the Jamtal hut is similar to sample location G, no individual of this species was observed, neither in these plots nor elsewhere in the glacier foreland. Whether this lack of pine trees is an indication of a different climatic setting in the past, or just a coincidence, will be subject to further studies.

Primary succession on new ground is commonly reflected by an increase in species numbers and ground cover, at least until a certain point^22,27^ (see Fig. 5). To some degree, the trend line for species numbers displays a negative logarithmic behaviour, indicating a kind of saturation in species establishment during succession, most likely due to inter- and intraspecific competition^22,28,29^. Also, the trend line for ground cover shows this effect towards the older sites. The early decades, however, behave more positively logarithmic, i.e., ground cover values lag slightly behind, despite a swift increase in species numbers during the pioneer and early successional stages (see Fig. 5).

Almost all species encountered within the glacier foreland are anemochorous (i.e. wind dispersed) taxa which are carried into the glacier foreland primarily by mountain-valley wind systems, even from lower elevations^30,31^. Despite good seed rain, immediate establishment of plants is not guaranteed for all diaspores reaching the glacier foreland, either due to harsh site conditions (cold, drought, excessive water, periglacial processes, etc.^23^) or because particular taxa require a period of dormancy to enhance germination success^32^.

The observed increase of NDVI between 1985 and 2016 at the sample sites proves that the increase in ground cover and species numbers generally can also be found in the remote-sensing data (Fig. 5). As a hypothesis, thus, the NDVI could be interpreted to reflect both an increase in ground cover and in species number. A validation of this hypothesis would require more field truth data, preferably also from other glacier forelands. Mean NDVIs for sub-periods reveal increases in NDVI over time (Table S4), but it is not straightforward to interpret the biotic changes without field references.

Generally, a separation of ground cover and species numbers would benefit from a better spatial and spectral resolution, as the signal can be considered a mixed signal from the ground and different parts of the vegetation. The selection of sample sites for this study was done in the field, selecting stable and representative terrain. For an automatic exclusion of unstable sites, repeat high resolution LiDAR data could be an option, currently available only for very few sites worldwide. For a general view on the changes in glacier forelands, for example, in a hydrological model or for climate change adaption/disaster management, time series of NDVI have high potential, although further work on the data interpretation is desirable. Anyway, our first results from Jamtalferner derive glacier retreat rates from vegetation cover as well as increasing plant cover from remote sensing and look promising enough to encourage further studies on other sites.

## Material and Methods

### Historical length change measurements, glacier outlines and graphical representations of Jamtalferner

The length change measurements at Jamtalferner had been initiated in 1892 by Ignaz Lorenz, head of the mountain guides and, together with his brother Gottlieb Lorenz, keeper of the Jamtal hut (established in 1882) of the Alpine Club^18^. The length change records and other material of the Austrian Alpine Club document a continuous retreat of Jamtalferner since the beginning of the measurements^17^. Past glacier outlines are evident from historical maps as well as from the delineation of glacier perimeters and dated moraines in orthophotos, more recently also in LiDAR DEMs. For all Silvretta glaciers, the glacier outlines for the LIA maximum, for 1969, 1996, 2002, and for 2006 have been compiled within the Austrian glacier inventories [^3^ and references herein]. For this study, all potential sources of glacier information for Jamtalferner’s post-LIA development have been compiled for a comprehensive set of glacier outlines (see Table S5).

The earliest map indicating roughly the size and position of the Jamtalferner dates from 1774^33^, however, sufficient details for a valid delineation of the extent of Jamtalferner are only given from the second federal survey (1816–1821) onwards. The LIA moraines are indicated in the second federal survey map and the texture of the glacier foreland can be clearly distinguished from the vegetated area outside the LIA glacier margins. The map of the third federal survey (1869–1887) shows much more detail and contour lines indicate elevation. The scale of 1:28,800 for the second survey and 1:25,000 for the third survey, in conjunction with the absence of local reference points, nevertheless increases the uncertainty in georeferencing these large-scale maps, so that we only used local maps for the reconstruction of area changes and glacier margins (see below). The same is true of the regional map of Trentinaglia-Telvenburg^24^, and the map of Ziegler^34^. In the early 20^th^ century, local maps of Jamtal and Jamtalferner were compiled by Haug^35^ and Greim^20,36–39^. They used a local coordinate systems and scales of 1:25,000 and 1:10,000, respectively, where pass points suitable for co-registration to contemporary coordinate systems can be identified. The map of the Swiss Alpine Club (SAC) of 1898^40^ shows a similar terminus position as the maps of Greim for 1898 and 1895/97, but a broader glacier tongue than displayed in the maps of Haug and Greim.

The various sources of information of different stages of cartographic details, techniques and resolutions made it necessary to first co-register the maps to the Austrian coordinate system Gauß-Krüger M28. The number and quality of reference points largely depend on source type, so that each type is described in detail below. The historical maps of the glacier tongue of^20,36,37^ use a local coordinate system. As only few reference points are marked in the maps, the maps have been georeferenced only, but not reprojected. As the spatial extent of the glacier tongue on the maps is small, and the information on control points is sparse, the error of the position of the glacier tongue from georeferencing errors can be put at about 200 m. As we have no indication on how many measured points were used for the construction of the terminus position, general assumptions as described by Brunner and Welsh^41^ or Haggrén *et al*.^42^ can be used for a rough estimate. For larger-scale maps, like that of Haug^35^, with more control points a higher accuracy in georeferencing can be assumed. The total RMS error of the georeferencing with 10 control points was 188 m. For the years between 1954 and 2015, the position of the glacier terminus was derived from orthophotos taken by the federal government (with exception of the 2006 glacier outline, which was mapped from airborne LiDAR data). The spatial resolution increases with time to 0.5 m. Glacier margins were mapped manually in ESRI ArcMap 10.6.1.9270. LiDAR DEMs were used for glacier delineation by calculating hill shades, which allow distinguishing smooth glacier surfaces from rougher periglacial areas^43^. Including maps of volume change allowed tackling debris-covered parts of the glacier as well. The errors are less than 4 m for 80% of the glacier boundary, as revealed by ground truthing^43^, and ±1.5% for the total glacier area, when mapping the glacier boundary would serve to calculate the area of a glacier.

### Vegetation sampling along a chronosequence

Vegetation sampling was performed by a chronosequence approach^44^, inferring a temporal sequence of vegetation development by spatially different sample sites of more or less precisely identified site age. Altogether nine stages of time since deglaciation were surveyed (A: 1–2 yrs.; B: 7 yrs.; C: 15 yrs.; D: 25 yrs.; E: 55 yrs.; F: 70 yrs.; G: 90 yrs.; H: 120 yrs.; J: 150 yrs). Each stage is represented by three 10 m^2^ sample plots (2 × 5 m; denoted: I, II, III; to avoid confusion sample-label I is missing) of “mean” site conditions (i.e. no wind-exposed knolls with drier conditions or topographical depressions with above-average snow cover duration). Vegetation sampling records ground cover of all vascular plants, as well as structural measures, such as life form composition (acc. to Raunkiaer^45^) and dispersal biology types (acc. to Müller-Schneider^46^) of the species present. The taxonomy of vascular plant species follows Fischer *et al*.^47^. Mosses are sampled as species-group (i.e. not differentiated to species level). Sampling took place m^2^-wise with the smallest unit being 0.01% ground cover (i.e. 1 cm × 1 cm on a 1m^2^-subplot). Raw data were subsequently converted to mean ground cover values and total number of species per 10 m^2^ sample site. For each sample site, elevation (by altimeter) and amount of coarse rocks (by visual assessment of coarse rocks >6 cm in%) are recorded as environmental variables.

Data analyses applied standard uni- and multivariate statistical procedures. As primary succession in glacier forelands commonly starts with simple agglomerations of plants and subsequently becomes more and more complex^22,28,29,44^, a quantitative assessment of the vegetation development during succession after glacier retreat is achieved by recording changes in species numbers, ground cover (of singular species and in total) as well as lifeform composition at different temporal stages. Temporal trends of changing ground cover and species numbers are derived by non-linear regressions.

To detect gradual changes in species composition within large data sets, multivariate ordination procedures, which assume underlying gradients within the data set, are appropriate tools. Gradual floristic differences are calculated in a multidimensional ordination space by means of similarity relationships. Ordinations aim to reduce the number of dimensions, making complex datasets with many species and/or samples interpretable. Here, an unconstrained linear PCA is employed. Graphical display of an ordination analysis is by a two-dimensional scatter plot of samples. Explanatory variables are displayed as arrows, which point from the origin of ordinates in the direction where samples with above average values of the particular variable are located. The length of the arrows represents the relevance of the variable. Changes in groundcover and life form spectra during succession are superimposed by pie charts. The ordination analysis was performed with the software Canoco 4.5.

### Remote-sensing data and NDVI calculation

Landsat satellites, which came into operation in 1972, turned out to be very useful for mapping changes in glacier area^48^, snow cover^49^ and other surface properties. Spatial resolution is 30 m for spectral bands working between 450 and 2400 nm, for thermal infrared bands resolution is 120 m and 100 m for Landsat 5 and Landsat 8, respectively. We used 13 Landsat 5 TM and Landsat 8 OLI images to map the spectral reflectivity between August 1985 and August 2016 (Table S6) with wavelength ranges of the specific channels shown in Table S7.

Since the 1970s, the Normalized Difference Vegetation Index (NDVI) has been used to map vegetation from remote-sensing images comparing reflectivity in the near infrared and red band (e.g.^50,51^. The algorithm uses the fact that both bands are sensitive to chlorophyll. The NDVI is calculated for each pixel of the selected Landsat data according to Eq. 1.

1 $${\rm{NDVI}}=({\rm{NIR}}-{\rm{RED}})/({\rm{NIR}}+{\rm{RED}})$$

For this study, only acquisitions at clear sky and snow-free conditions over the area of interest were selected from the Landsat Collection 1 data (cf. Table S6). The data are provided by the U.S. Geological Survey as orthorectified images in UTM/WGS84 map projection. The generation of NDVI maps from Landsat data was performed with parts of ENVEO’s in-house developed modular software package for remote sensing data processing.

NDVI was already used to analyse long-term changes in greening of Alpine vegetation in the French Alps^52^, showing significant increases over the years 2000–2018, with maximum increases in rocky habitats. Carlson *et al*.^52^ analysed the NDVI grid without comparing to field data. Several studies confirm that, despite small differences in the band wavelengths, time series of NDVI extracted from various Landsat sensors can reliably map vegetation changes [e.g.^53–55^]. Chlorophyll and thus NDVI show seasonal variability (e.g.^56^). To map long-term changes, we must therefore distinguish between seasonal variability and long-term changes in NDVI. In this study, only snow-free images in August were analysed, assuming that the comparison of remote-sensing data from the same month in different years shows long-term changes only. To detect changes in sparse vegetation, NDVI differencing performs better than classification^57^. The size of the sample plots is small compared to Landsat pixel size, so, basically, we can presume that, even in cases of uncertainties in georeferencing of a few metres, the field plot is still located within the corresponding Landsat grid cell.

## Supplementary information

Supplementary Material

## Data Availability

Data used within this study are available at Pangaea: https://issues.pangaea.de/browse/PDI-20733 Geodata (DEMs and ortophotos) used for the analysis are provided by the Federal Government of Tyrol within the open data initiative under CC BY 4.0 licence (source: Land Tirol - data.tirol.gv.at).

## References

[1] IPCC. Climate Change 2013: The Physical Science Basis. Contribution of Working Group I to the Fifth Assessment Report of the Intergovernmental Panel on Climate Change (eds Stocker, T. F. *et al*.). Cambridge University Press, Cambridge, United Kingdom and New York, NY, USA (2013).

[2] Fischer A (2015). Tracing glacier changes in Austria from the Little Ice Age to the present using a lidar-based high-resolution glacier inventory in Austria. The Cryosphere.

[3] Zemp M (2015). Historically unprecedented global glacier decline in the early 21st century. J Glaciol.

[4] Brighenti (2019). Ecosystem shifts in Alpine streams under glacier retreat and rock glacier thaw: A review. Sci Total Environ.

[5] Fountain AG (2012). The disappearing cryosphere: impacts and ecosystem responses to rapid cryosphere loss. Bioscience.

[6] Milner AM (2017). Glacier shrinkage driving global changes in downstream systems. Proc. Natl. Acad. Sci..

[7] Zemp M, Haeberli W, Hoelzle M, Paul F (2006). Alpine glaciers to disappear within decades?. Geophys. Res. Lett..

[8] Huss M (2017). Toward mountains without permanent snow and ice. Earth’s Future.

[9] Auer I (2007). HISTALP – Historical instrumental climatological surface time series of the greater Alpine region 1760–2003. Int. J. Climatol..

[10] Fischer, A. *et al*. What Future for Mountain Glaciers? Insights and implications from long-term monitoring in the Austrian Alps. In: *Developments in Earth Surface Processes vol 21* (eds Greenwood, G. B. & Shroder, J. F.), 325–382 (2016).

[11] Isotta FA (2014). The climate of daily precipitation in the Alps: development and analysis of a high‐resolution grid dataset from pan‐Alpine rain‐gauge data. Int J Climatol..

[12] Reitmaier T (2013). Alpine Archäologie in der Silvretta. Archäologie der Schweiz.

[13] Dietre B (2014). Palaeoecological evidence for Mesolithic to Medieval climatic change and anthropogenic impact on the Alpine flora and vegetation of the Silvretta Massif (Switzerland/Austria). Quaternary International.

[14] Patzelt, G. *Gletscher. Klimazeugen von der Eiszeit bis zur Gegenwart* (Hatje-Cantz, Berlin, in press).

[15] Fischer A, Markl G (2009). Mass balance measurements on Hintereisferner, Kesselwandferner and Jamtalferner 2003 to 2006: database and results. Zeitschrift für Gletscherkunde und Glazialgeologie.

[16] Rutzinger, M., Moran, A., Fischer, A. & Groß, G. Klimawandel und Klimageschichte -Die Gletscher der Silvretta unter wandelnden Klimabedingungen. In: *Silvretta Historica*, *Sonderband zur Montafoner Schrifenreihe 20* (ed. Kaspar, M., Heimatschutzverein Montafon, Wien) (2013).

[17] Groß, G. Gletscher- und Klimaentwicklung rund um den Piz Buin. In: *Mythos* Biz Buin (ed. Kasper, M.), 21–48 (Haymon, Innsbruck, 2015).

[18] Groß Günther (2017). Die Geschichte der Gletscherbeobachtung und -messung in den Österreichischen Alpen. Gletscher im Wandel.

[19] Vorndran G (1968). Untersuchungen zur Aktivität der Gletscher -dargestellt an Beispielen aus der Silvrettagruppe. Schriften des Geographischen Instituts der Universität Kiel.

[20] Greim G (1934). Studien aus dem Paznaun III. Jamferner und Jambach von 1901–1921. Gerlands Beiträge zur Geophysik.

[21] Cannone N, Diolaiuti G, Guglielmin M, Smiraglia C (2008). Accelerating climate change impacts on alpine glacier forefield ecosystems in the European Alps. Ecol Appl.

[22] Fickert, T. *Glacier Forelands -Unique Field Laboratories for the Study of Primary Succession of Plants*. In: Glaciers Evolution in a Changing World (ed. Godone, D.), InTech Open, 125–146 (2017).

[23] Fickert T, Grüninger F (2018). High-speed colonization of bare ground – Permanent plot studies on primary succession of plants in recently deglaciated glacier forelands. Land Degrad Dev.

[24] Trentinaglia-Telvenburg, J. *Das Gebiet der Rosanna und Trisanna* (Carl Gerold’s Sohn, Wien, 1875).

[25] Greim G (1906). Studien aus dem Paznaun II, Der Jamtalferner bis 1897. Gerlands Beiträge zur Geophysik.

[26] Klebelsberg R (1913). von. Das Vordringen der Hochgebirgsvegetation in den Tiroler Alpen—eine alpin-pflanzengeographische Studie. Österreichische Botanische Zeitschrift.

[27] Andreis C, Caccianiga M, Cerabolini B (2001). Vegetation and environmental factors during primary succession on glacier forelands. Plant Biosyst.

[28] Schumann K, Gewolf S, Tackenberg O (2016). Factors affecting primary succession of glacier foreland vegetation in the European Alps. Alpine Bot.

[29] Erschbamer B, Caccianiga MS (2017). Glacier Forelands: Lessons of Plant Population and Community. Development. Progress in Botany.

[30] Erschbamer B, Kneringer E, Niederfriniger Schlag R (2001). Seed rain, seed bank, seedling recruitment, and survival of seedlings on a glacier foreland in the Central Alps. Flora.

[31] Tackenberg O, Stöcklin J (2008). Wind dispersal of alpine plant species: A comparison with lowland species. J Veg Sci.

[32] Schwienbacher E, Erschbamer B (2001). Longevity of seeds in a glacier foreland of the Central Alps – A burial experiment. Bulletin of the Geobotanical Institute ETH.

[33] Anich, P. & Hueber, B. Tyrolis sub felici regimine Mariae Theresiae Rom. Imper. Aug. chorographice delineata a Petro Anich et Blasio Hueber Colonis oberperfussianis Curante Ignat. Weinhart Profess. Math. in Univers. Oenipontana. Aeri incisa á Ioa. (Erneste Mansfeld, Viennae, 1774).

[34] Ziegler, J. M. Unter-Engadin - *Karte des Unter-Engadins: mit den nördlich, östlich u. südlich angrenzenden Theilen von Vorarlberg, Tyrol und Veltlin 1:50 000* (Wurster, Randegger & Co, Winterthur, 1869).

[35] Haug, E. Umgebung der Jamtalhütte nach photogrammetrischen Aufnahmen von E. Haug, Stuttgart. Maßstab 1:25.000. Supplement to Hess, H., *Zeitschrift des Deutschen und Österreichischen Alpenvereins* **40** (Verlag des Deutschen und Österreichischen Alpenvereins, München, 1909).

[36] Greim G (1903). Studien aus dem Paznaun I. Gerlands Beiträge zur Geophysik.

[37] Greim G (1904). Studien aus dem Paznaun (Die Ergebnisse der Messungen des Jambaches 1903). Meteorologische Zeitschrift.

[38] Greim, G. Studien aus dem Paznaun IV, *Gerlands Beiträge zur Geophysik* **48**, (1936).

[39] Greim G (1943). Beobachtungen am Jamtalferner. Petermanns Geographische Mitteilungen.

[40] SAC Silvretta-Muttler-Lischanna, Excursionskarte des Schweizer Alpen-Club-Pro 1898, 1:50.000 Supplement to *Jahrbuch des SAC* **33 (**Kümmerly & Frey, Bern 1898).

[41] Brunner, K. & Welsch, W. Untersuchungen zur Georeferenzierung von Alpenvereinskarten. *Wissenschaftliche Alpenvereinshefte* **34** (Österreichischer Alpenverein, Innsbruck, 2001).

[42] Haggrén H, Mayer C, Nuikka M, Rentsch H, Peipe J (2007). Processing of old terrestrial photography for verifying the 1907 digital elevation model of Hochjochferner Glacier. Zeitschrift für Gletscherkunde und Glazialgeologie.

[43] Abermann J, Kuhn M, Fischer A (2011). Climatic controls of glacier distribution and changes in Austria. Annals of Glaciology.

[44] Matthews, J. A. *The Ecology of recently-deglaciated terrain. A geoecological approach to glacier forelands and primary succession* (Cambridge University Press, Cambridge, 1992).

[45] Raunkiaer, C. *The life‐forms of plants and statistical plant geography* (Oxford University Press, Oxford, 1934).

[46] Müller-Schneider, P. *Verbreitungsbiologie der Blütenpflanzen Graubündens* (Veröffentlichungen des Geobotanischen Instituts der Eidg. Techn. Hochschule, Stiftung Rübel, Zürich, 1986),

[47] Fischer, M. A., Adler, W. & Oswald, K. *Exkursionsflora für Österreich, Liechtenstein und Südtirol*. Land Oberösterreich, Biologiezentrum der OÖ Landesmuseen, Linz (2005).

[48] Zemp M (2011). Summary of international glacier monitoring summit. The Earth Observer.

[49] Rott H, Markl G (1989). Improved snow and glacier monitoring by the Landsat Thematic Mapper. ESA SP.

[50] Colwell JE (1974). Vegetation canopy reflectance. Remote Sensing of Environment.

[51] Tucker CJ (1979). Red and photographic infrared linear combinations for monitoring vegetation. Remote Sensing of Environment.

[52] Carlson B (2017). Observed long-term greening of alpine vegetation—a case study in the French Alps. Environ. Res. Lett..

[53] Xu D, Guo X (2014). Compare NDVI Extracted from Landsat 8 Imagery with that from Landsat 7 Imagery. American Journal of Remote Sensing.

[54] Mandanici E, Bitelli G (2016). Preliminary Comparison of Sentinel-2 and Landsat 8 Imagery for a Combined Use. Remote Sensing.

[55] Zhang HK (2015). Characterization of Sentinel-2A and Landsat-8 top of atmosphere, surface, and nadir BRDF adjusted reflectance and NDVI differences. Remote Sensing of Environment.

[56] Schmid H, Karnieli A (2000). Remote sensing of the seasonal variability of vegetation in a semi-arid environment. J Arid Environ.

[57] Pu R (2008). Using classification and NDVI differencing methods for monitoring sparse vegetation coverage: a case study of saltcedar in Nevada, USA. Int J Remote Sens.

