# Author Correction: Vegetation dynamics in Alpine glacier forelands tackled from space

**DOI:** 10.1038/s41598-020-64607-y

**Published:** 2020-05-01

**Authors:** Andrea Fischer, Thomas Fickert, Gabriele Schwaizer, Gernot Patzelt, Günther Groß

**Affiliations:** 1 Institute for Interdisciplinary Mountain Research, Austrian Academy of Sciences, Technikerstr. 21a, 6020 Innsbruck, Austria; 2 Faculty of Arts and Humanities, University of Passau, Innstraße 40, 94032 Passau, Germany; 3 ENVEO IT GmbH, Fürstenweg 176, 6020 Innsbruck, Austria; 4 Patscher Strasse 20, 6080 Igls, Austria; 5 Oberrain 205, 6721 Thüringerberg, Austria

Correction to: *Scientific Reports* 10.1038/s41598-019-50273-2, published online 26 September 2019

In this Article, the Data availability section contains an incorrect link:

“Data used within this study are available at Pangaea: https://issues.pangaea.de/browse/PDI-20733 Geodata (DEMs and ortophotos) used for the analysis are provided by the Federal Government of Tyrol within the open data initiative under CC BY 4.0 licence (source: Land Tirol - data.tirol.gv.at).”

should read:

“Data used within this study are available at Pangaea: 10.1594/PANGAEA.902545 Geodata (DEMs and ortophotos) used for the analysis are provided by the Federal Government of Tyrol within the open data initiative under CC BY 4.0 licence (source: Land Tirol - data.tirol.gv.at).”

