# Supplementary material for: Vegetation dynamics in Alpine glacier forelands tackled from space
Source: Sci Rep. 2019 Sep 26;9:13918. doi: 10.1038/s41598-019-50273-2 (PMC6763459; doi:10.1038/s41598-019-50273-2)
Supplement: Supplementary file 1 — Supplementary Material [file 41598_2019_50273_MOESM1_ESM.pdf]

Supplementary material

Vegetation dynamics in Alpine glacier forelands tackled from space

Andrea Fischer (1)*, Thomas Fickert (2), Gabriele Schwaizer (3), Gernot Patzelt (4), Günther Groß (5)

(1) Institute for Interdisciplinary Mountain Research, Austrian Academy of Sciences,

Technikerstr. 21a, 6020 Innsbruck, Austria.

(2) Faculty of Arts and Humanities, University of Passau, Innstraße 40, 94032 Passau, Germany.

(3) ENVEO IT GmbH, Fürstenweg 176, 6020 Innsbruck, Austria.

(4) Patscher Strasse 20, 6080 Igls

(5) Oberrain 205, 6721 Thüringerberg

* Correspondence should be addressed to A.F. (email: andrea.fischer@oeaw.ac.at)

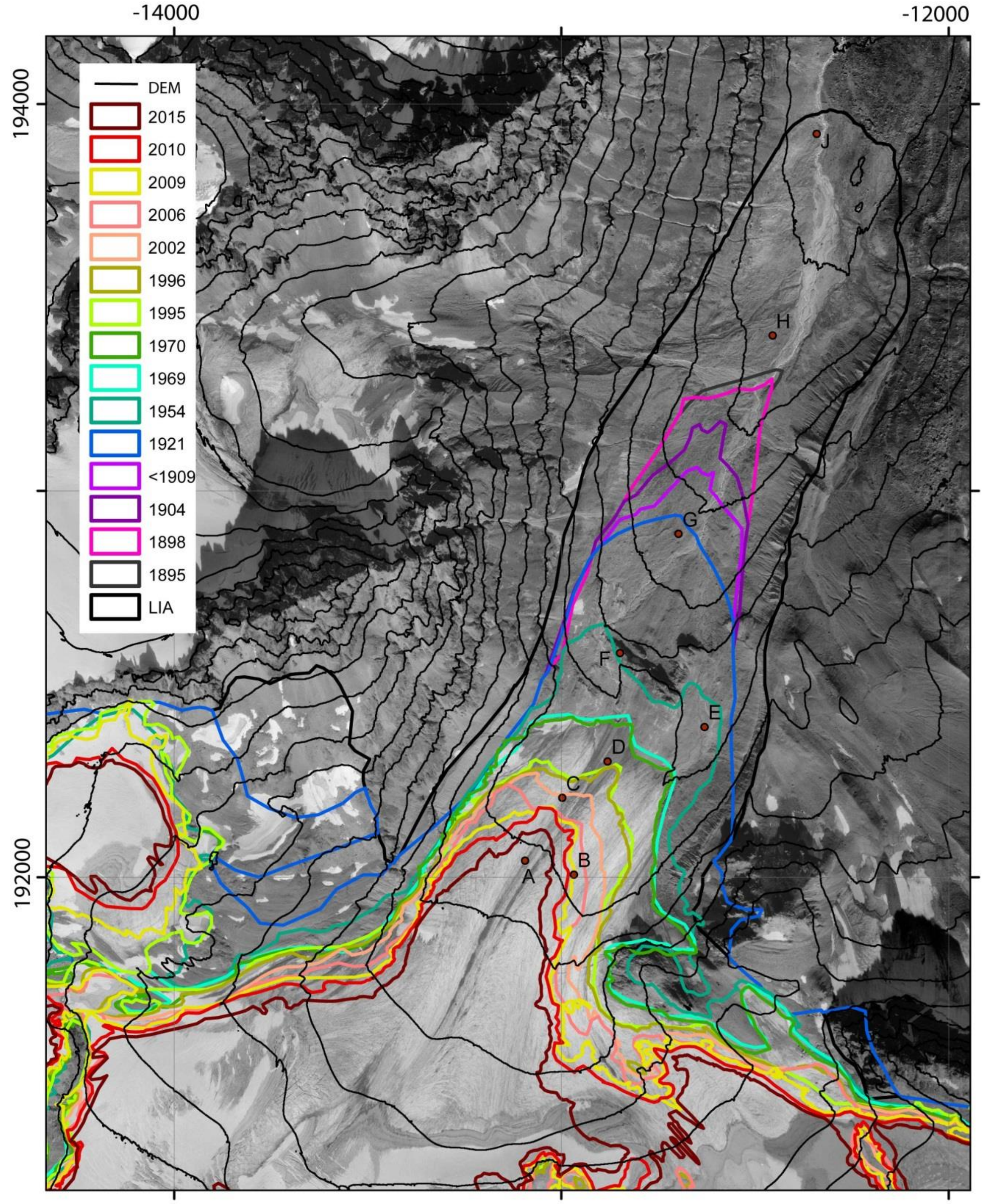

Fig. S1: Extent of Jamtalferner in various years between LIA maximum and 2015, as well as contour lines (DEM of 2006) superimposed on an orthophoto from 1970 (source: Land Tirol - data.tirol.gv.at CC BY 4.0).

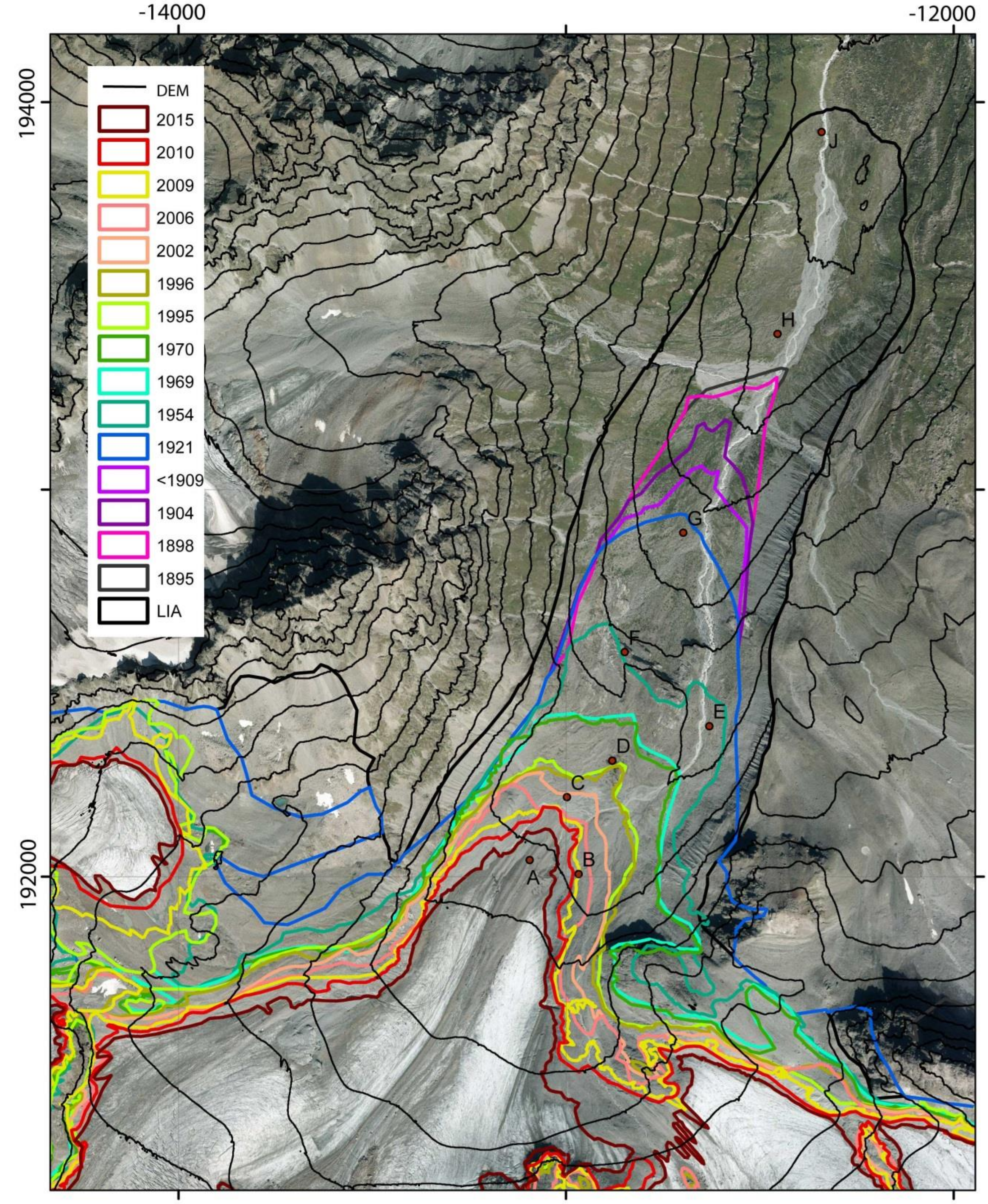

Fig. S2: Extent of Jamtalferner in various years between LIA maximum and 2015, as well as contour lines (DEM of 2006) superimposed on an orthophoto from 2015 (source: Land Tirol - data.tirol.gv.at CC BY 4.0).

Table S1: Monthly, seasonal and annual means of air temperature and precipitation sums at the Galtür station (1587 m, 1951-2000) and precipitation at the rain gauge at the tongue of Jamtalferner (2400 m, 1989-2017), provided by the Hydrographical Service of the Federal Government of Tyrol. % of Galtür: percentage of precipitation at the glacier tongue compared to precipitation measured in Galtür. See also the insert in Figure 1.

Galtür	Oct	Nov	Dec	Jan	Feb	Mar	Apr	May	Jun	Jul	Aug	Sep	May-Sep	Oct-Apr	year
Temperature in °C	3.9	-1.7	-5.0	-5.9	-5.0	-2.2	1.3	6.3	9.5	12	11.2	8.2	9.4	-2.1	2.7
Precipitation in mm	60	64	62	66	57	60	54	81	123	147	142	97	590	423	1013
Jamtalferner	Oct	Nov	Dec	Jan	Feb	Mar	Apr	May	Jun	Jul	Aug	Sep	May-Sep	Oct-Apr	year
Precipitation in mm	102	97	98	94	93	93	90	120	183	195	205	137	840	667	1507
% of Galtür	170	151	159	142	163	155	167	148	149	133	144	141	142	158	149

Table S2. Fossil trees (*Pinus cembra*) found close to the Jamtal hut with lab code and results.

sample ID	laboratory ID	altitude	14C age BP	calibrated age BC (2σ)
FT-1	VRI-1990	2290	4630±60	3631-3116
FT-2	VRI-1991	2240	4550±60	3500-3020

Table S3: Species list with information on mean ground cover at the plots, as well as family and life form affiliation (Th = therophytes, G = geophytes, H herb, H gram = herbaceous and graminoid hemicryptophytes, Ch = chamaephytes, NaPh, MakPh = nano- and macrophanerophytes, lichen, moss) and dispersal mode.

Chronosequence Jamtalferner												
		time since deglaciation in a		1-2	7	15	25	55	70	90	120	150
		elevation in m a.s.l.		2450	2430	2420	2400	2390	2320	2250	2160	2120
		number of species (incl. mosses & lichens)		14	27	22	22	34	31	42	43	47
		number of vascular plant species		13	26	20	20	32	29	39	40	42
		mean total ground cover		0.35	4.24	10.15	13.35	42.80	57.33	78.72	72.88	77.13
		mean cover vascular plants		0.31	2.32	2.39	9.88	29.45	44.06	54.17	46.56	63.68
				mean groundcover (out of three sample sites per level)								
species	family	life form	dispersal mode	A	B	C	D	E	F	G	H	J
Tussilago farfara	Asteraceae	G	trichometeorochorous	0.0040	0.0033							
Epilobium anagallidifolium	Onagraceae	H herb	trichometeorochorous	0.2467	0.0270	0.0097	0.0220					
Sagina saginoides	Caryophyllaceae	H herb	boleochorous	0.0087	0.1477	0.0017		0.0017	0.0003			
Cerastium uniflorum	Caryophyllaceae	Ch	boleochorous	0.0057	0.7050	0.3240	0.5867	0.0323	0.0433			
Geum reptans	Rosaceae	H herb	trichometeorochorous	0.0007	0.1710	0.0407	0.0833	0.0917	0.6303			
Veronica alpina	Veronicaceae	H herb	boleochorous	0.0017	0.0133	0.0453	0.0107	0.0010	0.0153			
Doronicum clusii ssp. clusii	Asteraceae	H herb	trichometeorochorous	0.0033		0.0627		0.0873	0.5793	0.0067		
Epilobium angustifolium	Onagraceae	H herb	trichometeorochorous	0.0040	0.0167					0.0333		
Poa laxa	Poaceae	H gram	trichometeorochorous	0.0070	0.5023	0.1123	0.7333	0.5200	0.3883	0.4113	0.4167	
Hieracium intybaceum	Asteraceae	H herb	trichometeorochorous	0.0100							0.6733	
Sedum alpestre	Crassulaceae	Ch	boleochorous	0.0073	0.0050	0.0243	0.0417	0.0087	0.0143	0.0030	0.0123	
Saxifraga bryoides	Saxifragaceae	Ch	boleochorous	0.0017	0.2057	0.8517	2.8933	2.2603	0.5383	0.3513	0.0273	0.0133
Gnaphalium supinum	Asteraceae	H herb	trichometeorochorous	0.0063	0.0070	0.0530	0.0323	0.0013	0.0323	0.0027		0.0037
moss		moss		0.0380	1.9187	7.7500	3.4633	9.7667	13.1333	23.7000	24.4333	12.2333
Festuca pumila	Poaceae	H herb	pterometeochorous		0.0100							
herbaceous Asteraceae undetermined	Asteraceae	H herb	???		0.0003							
Arenaria biflora	Caryophyllaceae	Ch	boleochorous		0.0007							
Saxifaraga exarata ssp. exarata	Saxifragaceae	Ch	boleochorous		0.0017		0.0167					
Arabis alpina	Brassicaceae	Ch	boleochorous		0.0077		0.0020					
Silene exscapa	Caryophyllaceae	Ch	boleochorous		0.0067			0.0167				
Salix reticulata	Salicaceae	Ch	trichometeorochorous		0.0133			0.0067				
Oxyria digyna	Polygonaceae	H herb	pterometeochorous		0.0067		0.0833	0.0017	0.0073			
Dryopteris expansa	Dryopteridaceae	H herb			0.0043	0.0940	0.1807		0.0367	0.0017	0.0023	
Salix herbacea	Salicaceae	Ch	trichometeorochorous		0.0133			0.8353	2.3140	1.0333	0.0067	0.1430
Cardamine resedifolia	Brassicaceae	H herb	ballochorous		0.0003	0.0153	0.0317	0.0007	0.0027	0.0007	0.0013	0.0003
Salix retusa	Salicaceae	Ch	trichometeorochorous		0.0167	0.0183		2.6100	1.5173	3.0333	0.1333	0.2600
Epilobium fleischeri	Onagraceae	H herb	trichometeorochorous		0.2670							0.0500
Salix hegetschweileri	Salicaceae	NaPh	trichometeorochorous		0.0533		2.5667	0.2333		5.3000	0.5667	0.5500
Leucanthemopsis alpina	Asteraceae	H herb	boleochorous		0.1180	0.4547	1.5307	0.1140	0.8893	0.1160	0.0087	0.0013
Poa alpina	Poaceae	H gram	trichometeorochorous (ps.-vivipar)			0.0170			0.2123			
Adenostyles alliariae	Asteraceae	H herb	trichometeorochorous			0.0500				0.3073		
Gymnocarpium dryopteris	Woodsiaceae	G				0.0003					0.0333	
Stereocaulon alpinum	Stereocaulaceae	Lichen				0.0117	0.0003	3.5747	0.1317	0.6510	1.4167	0.3877
Agrostis alpina	Poaceae	H gram	pterometeochorous			0.1340	0.0033	0.0227	0.0300	0.0873	0.4557	0.1567
Senecio incanus spp. carniolicus	Asteraceae	H herb	trichometeorochorous			0.0453	0.9803	0.3127	1.1580	0.3750	0.4407	0.2280
Leontodon hispidus	Asteraceae	H herb	trichometeorochorous			0.0333	0.0457	0.0370	0.0350	0.0850	3.2233	1.8123
Campanula scheuchzeri	Campanulaceae	H herb	boleochorous				0.0400	0.0003		0.0683		0.0150
Phyteuma globulariifolium	Campanulaceae	H herb	boleochorous					0.0003				
Primula hirsuta	Primulaceae	H herb	boleochorous					0.0067				
Saxifraga oppositifolia	Saxifragaceae	Ch	boleochorous					0.0033				
Luzula alpina	Juncaceae	H gram	stomatochorous (ants)					0.0003		0.0033		
Myosotis alpestris	Boraginaceae	H herb	boleochorous					0.0020	0.0323	0.1033	0.0167	
Luzula alpinopilosa	Juncaceae	H gram	stomatochorous (ants)					0.0500	1.5000	1.3950	0.2167	
Hieracium alpinum	Asteraceae	H herb	trichometeorochorous					0.0013	0.0013	0.0367	0.1563	0.1590
Vaccinium gaultherioides	Ericaceae	Ch	endochorous					0.8667				4.9300
Salix helvetica	Salicaceae	NaPh	trichometeorochorous					21.3340	24.3907	22.2000	8.4333	1.4667
Euphrasia minima	Orobanchaceae	Th	boleochorous					0.0203	0.0003	0.2633	0.0850	0.0177
Homogyne alpina	Asteraceae	H herb	trichometeorochorous					0.0067		0.0003	0.0120	0.1203
Arabis caerulea	Brassicaceae	H herb	boleochorous						0.0003			
Phleum commutatum	Poaceae	H gram	pterometeochorous						0.1767			
Cirsium spinosissimum	Asteraceae	H herb	trichometeorochorous						0.2447		0.0500	
Lotus corniculatus	Fabaceae	H herb	boleochorous						1.5370		0.3807	0.7000
Salix glaucosericea	Salicaceae	NaPh	trichometeorochorous						6.0667	14.2500	15.8367	13.5333
Trifolium pallescens	Fabaceae	H herb	pterometeochorous							0.3600		
Trifolium pratense ssp. nivale	Fabaceae	H herb	pterometeochorous							0.5667		
Saxifraga paniculata	Saxifragaceae	Ch	boleochorous							0.0007		
Empetrum hermaphroditum	Ericaceae	Ch	endochorous							0.1500	0.0667	10.7667
Loiseleuria procumbens	Ericaceae	Ch	boleochorous							0.8333		2.4333
Lycopodium alpinum	Lycopodiaceae	Ch								0.0033		0.0100
Bartsia alpina	Orobanchaceae	H herb	boleochorous							0.1167	0.0133	0.4043
Cetraria islandica	Pameliaceae	Lichen								0.1917	0.4667	3.7867
Rhododendron ferrugineum	Ericaceae	NaPh	boleochorous							3.1875	7.3833	8.9667
Vaccinium myrtillus	Ericaceae	Ch	endochorous							0.0500		1.4000
Huperzia selago	Lycopodiaceae	Ch								0.0993	0.0317	0.0180
Campanula barbata	Campanulaceae	H herb	boleochorous							0.7133	1.2000	0.1770
Pyrola rotundifolia	Ericaceae	H herb	cystometeochorous							0.1450	0.0200	0.1317
Larix decidua	Pinaceae	MakPh	pterometeochorous							0.3333	7.0333	2.5000
Hieracium bifidum	Asteraceae	H herb	trichometeorochorous							0.0833	0.0167	
Solidago virgaurea ssp. minuta	Asteraceae	H herb	trichometeorochorous								1.1100	
Gentiana nivalis	Gentianaceae	Th	boleochorous								0.0050	
Alchemilla glabra	Rosaceae	H herb	pterometeochorous								0.0017	
Thesium alpinum	Santalaceae	H herb	stomatochorous (ants)								0.0003	
Orthilia secunda	Ericaceae	Ch	cystometeochorous								0.1040	0.0033
Anthyllis vulneraria ssp. alpicola	Fabaceae	H herb	cystometeochorous								0.0340	0.0033
Potentilla aurea	Rosaceae	H herb	boleochorous								0.0017	0.0007
Sempervivum montanum ssp. montanum	Crassulaceae	Ch	boleochorous								0.0033	0.0600
Agrostis agrostifolia	Poaceae	H gram	pterometeochorous								0.6000	0.1900
Anthoxantum alpinum	Poaceae	H gram	trichometeorochorous									4.0807
Poa variegata	Poaceae	H gram	trichometeorochorous									0.4167
Phyteuma hemisphericum	Campanulaceae	H herb	boleochorous									0.0010
Gentiana punctata	Gentianaceae	H herb	boleochorous									0.2000
Festuca nigricans	Poaceae	H herb	pterometeochorous									1.1500
Cladonia spec.	Cladoniaceae	Lichen										0.0733
Alectoria ochroleuca	Pameliaceae	Lichen										0.0650
Carex curvula	Cyperaceae	H gram	cystometeochorous									0.0050
Picea abies	Pinaceae	MakPh	pterometeochorous									0.2667
Calluna vulgaris	Ericaceae	NaPh	cystometeochorous									3.2400

Table S4: NDVI 2016, as well as means and changes for two periods (1985-1991, 2009) at the sample sites with altitude. * glacier area at that date.

Ice-free years	Sample	Altitude	NDVI mean 1985-1991	NDVI mean 2009	NDVI 85_91-2009	NDVI 2016
1	A	2464	*	*	0.09	-0.03
7	B	2430	*	*	0.12	0.00
15	C	2409	*	0.03	0.08	0.11
25	D	2405	0.01	0.13	0.11	0.21
55	E	2389	0.16	0.29	0.13	0.40
70	F	2384	0.16	0.29	0.13	0.40
90	G	2271	0.27	0.29	0.02	0.35
120	H	2177	0.32	0.40	0.08	0.46
150	J	2123	0.36	0.43	0.07	0.50
average			0.11	0.20	0.09	0.27

Table S5: Maps and DEMs of Jamtalferner with source and date. GI1,2,3: part of the glacier inventory. BW: Black and white images. * The LIA maximum state has been reported for 1864, based on a conversation of Greim with Gottlieb Lorenz (1844-1911), who was a shepherd in Jamtal in his 20s. This oral reported date is not consistent with other literature, for example, Richter's observation about the contact of Jamtalferner and Totenfeldferner and older maps. Therefore, this date must be considered uncertain. TIRIS: orthophotos of surveys of the federal government of Tyrol.

Year	Type	Source	Remarks
2015	orthophoto	TIRIS	
2010	orthophoto	TIRIS	
2006	LiDAR	TIRIS	GI3
2002	orthophoto	TIRIS	
2002	orthophoto		BW GI2
1996	orthophoto		GI2
2009	orthophoto	TIRIS	
1995	orthophoto	TIRIS	BW
1970	orthophoto	TIRIS	BW
1969	orthophoto		BW GI1
1954	orthophoto	TIRIS	BW
1921	map	[20]	1:15,000
not specified	map	[35]	1:25,000
1909	map	[20]	1:15,000
1904	map	[20]	1:15,000
1898	map	[20]	1:15,000
?	map	[40]	1:50,000
1895/1897	map	[25]	1:10,000
1888	point		
1870	point		
	map	[34]	1:50,000
~1864*	LIA moraine	[20]	GI LIA

Table S6: List of remote-sensing images used in the study.

Satellite sensor	Date	Path/Row
Landsat 5 TM	19850813	193/027
Landsat 5 TM	19900802	194/027
Landsat 5 TM	19900811	193/027
Landsat 5 TM	19910830	193/027
Landsat 5 TM	19980808	194/027
Landsat 5 TM	20040801	193/027
Landsat 5 TM	20060823	193/027
Landsat 5 TM	20070826	193/028
Landsat 5 TM	20090806	194/027
Landsat 5 TM	20090815	193/027
Landsat 5 TM	20090831	193/028
Landsat 8 OLI	20130801	194/027
Landsat 8 OLI	20150731	193/027
Landsat 8 OLI	20160825	194/027

Table S7: Spectral bands used for this study with wavelength range and spatial resolution for the sensors in Table 4.

	Red		NIR	
Satellite sensor	wavelength range (nm)	Resolution (m)	wavelength range (nm)	Resolution (m)
Landsat 5 TM	630-690	30	760-900	30
Landsat 8 OLI	636-673	30	851-879	30
